# Mode of Anesthesia and Quality of Recovery After Breast Surgery: A Case Series of 100 Patients

**DOI:** 10.7759/cureus.13822

**Published:** 2021-03-11

**Authors:** Ganeshkrishna Nair, Danny J Wong, Edmund Chan, Tamara Alexander, Rajeev Jeevananthan, Amit Pawa

**Affiliations:** 1 Anaesthesia, Guy’s & St Thomas' National Health Service (NHS) Foundation Trust, London, GBR; 2 Anaesthesia, Guy's & St Thomas' National Health Service (NHS) Foundation Trust, London, GBR

**Keywords:** quality of recovery, breast surgery, regional anesthesia

## Abstract

Purpose: Regional anesthesia techniques may improve patient recovery beyond treating postoperative pain alone and may facilitate patients in their return to functional, psychological as well as emotional baselines. We hypothesized that the quality of recovery (QoR) experienced by patients following breast surgery was associated with the type of anesthesia received as well as the use of a regional anesthesia technique during surgery.

Methods: We performed a single-center prospective, observational cohort study of patients undergoing elective breast procedures (both cancer and non-cancer surgery).

Results: One hundred patients completed baseline QoR-15 questionnaires prior to surgery, of which 96 also completed QoR-15 questionnaires on postoperative day 1. The median (IQR) QoR-15 score at baseline was 133 (124-141), decreasing to 121 (106.75-136.25) on postoperative day 1. In multivariable linear regression analysis, paravertebral blocks (PVB) were associated with a 16.7 point higher overall QoR-15 score on postoperative day 1 compared to no block (95% Confidence Interval [CI]: 7.7-25.8, p<0.001); while the use of combination blocks was associated with a 21.8 point higher postoperative QoR-15 score compared to no block (95% CI: 12.8-30.8, p<0.001). PVB and combination blocks were further associated with better postoperative pain, physical comfort, physical independence and emotional state scores, compared with no block. The use of total intravenous anesthetic was not associated with differences in postoperative QoR-15 score versus volatile anesthetic, after covariate adjustment.

Conclusion: Breast surgery patients receiving PVB or a combination of regional blocks during surgery have higher postoperative QoR-15 scores, after adjustment for other factors.

## Introduction

Surgery for breast cancer is associated with moderate to severe pain and is a risk factor for persistent postsurgical pain. Regional anesthesia techniques have been employed to improve postoperative analgesia in an attempt to reduce the severity of acute pain and the incidence of chronic pain after breast surgery [1,2]. While thoracic epidurals and paravertebral blocks (PVB) are well-established regional anesthesia techniques used for breast surgery, they are associated with a number of potential complications, thus limiting their use. In recent years, newer techniques such as erector spinae plane (ESP), the mid-point transverse process to pleura (MTP), pectoralis plane block (PECS) and serratus anterior plane (SAP) block have increasingly been used for perioperative pain control in patients undergoing breast surgery in the hope that they provide safe, easy and effective alternatives to epidural and PVB analgesia [3-11].

There is evidence that the quality of recovery (QoR) may be improved with regional anesthesia techniques, as shown in studies using the QoR multidimensional assessment instrument [11,12]. In addition, regional anesthesia techniques may provide important improvements to patient recovery beyond the control of postoperative pain alone and may facilitate patients in their return to functional, psychological as well as emotional baselines.

We, therefore, hypothesized that the QoR experienced by patients following breast surgery may be associated with the type of anesthesia they received as well as the use of a regional anesthesia technique during their operations.

## Materials and methods

Our study was registered as a service evaluation and received institutional ethical waiver (Guy’s and St Thomas’ National Health Service [NHS] Foundation Trust service evaluation number: 10342). We report our findings according to the strengthening reporting of observational studies in epidemiology (STROBE) checklist [13]. We performed a prospective observational cohort study of patients undergoing breast surgery at our institution over a 10-month period (December 2019 to September 2020). Adult patients undergoing elective breast procedures (both cancer and non-cancer surgery) were included in the study. The surgical procedures performed included partial mastectomy with sentinel lymph node biopsy, simple mastectomy, mastectomy with sentinel lymph node biopsy, modified radical mastectomy, mastectomy with implant insertion and implant removal.

Patients completed the QoR-15 questionnaire prior to their operation on the morning of surgery during the anesthetic pre-assessment visit. The QoR-15 is a validated short-form postoperative QoR questionnaire that measures the QoR from a patient’s perspective and assesses multiple patient-centered outcomes, and has been advocated in ambulatory and day surgery scenarios [14-16]. The questionnaire has been recommended for use in perioperative clinical trials that evaluate patient comfort and pain after surgery [17]. The QoR-15 assesses patient-centered outcomes, grouped into five domains that are relevant markers of the quality of care achieved: pain, physical comfort, physical independence, psychological support and emotional state [18]. The 11-point numerical rating scale leads to a minimum score of 0 (very poor recovery) and a maximum score of 150 (excellent recovery). No modification to anesthetic technique was mandated as part of this observational study, and the conduct of anesthesia was according to the anesthesiologist’s routine practice. The choice of general anesthetic and mode of regional anesthesia technique used, if any, were recorded. Patients who did not receive a regional anesthesia block by the anesthesiologist received surgical infiltration of local anesthetic by the surgeon. The postoperative analgesic regimen consisted of regular oral paracetamol, ibuprofen (unless contraindicated) and oral opioids, as per the anesthesiologist’s usual practice.

The patients were followed-up the next day and completed a further QoR-15 questionnaire either in-person or over the telephone. Additionally, baseline patient characteristics and perioperative data including age, gender, ethnicity, Association of Anesthesiologists (ASA) physical status grade, body mass index (BMI), extent and type of surgery were collected. The extent and type of surgery were collected as free-text descriptions of the operation and were then categorized into severity grades depending on the extent of the surgical insult (minor, moderate, major/complex) by two of the authors (AP and DJNW) through consensus agreement.

We report descriptive statistics of the overall QoR-15 scores at baseline and on postoperative day 1 for the cohort, and for each of the five subdomains of QoR-15: pain, physical comfort, physical independence, psychological support and emotional state. A QoR-15 score of 118 is considered consistent with good recovery and the difference in QoR-15 that patients consider important - the minimal clinically important difference (MCID) has been previously estimated as 8 [19].

We then performed linear regression modeling of the overall QoR-15 score for postoperative day 1 against the type of general anesthetic and against the type of regional block received by the patient. To identify variables associated with better QoR after surgery, a multivariable linear regression model was constructed adjusting for the baseline QoR-15 score recorded preoperatively, and the following other covariates thought to be associated with recovery through confounding: age, ASA grade, surgical severity (minor, moderate, major/complex). We repeated the modeling for each of the five subdomains of QoR-15 as dependent (outcome) variables, against the same covariates as in our earlier model, to investigate whether the associated factors identified in the first multivariable model were similarly associated with all subdomains of recovery.

All analyses were performed in R version 3.5.2 (R Foundation for Statistical Computing, Vienna, Austria). Means and standard deviations are reported for normally or uniformly distributed data. Medians and interquartile ranges are reported for skewed distributions. For all hypothesis tests, two-tailed tests were used and a p-value of <0.05 was considered significant.

## Results

One hundred patients completed the baseline QoR-15 questionnaire prior to surgery, of which 96 also completed the QoR-15 questionnaire on postoperative day 1 (Table 1).

**Table 1:** Patient demographics. BMI = body mass index; ASA = Association of Anesthesiologists Physical Status grade; GA = general anesthetic; ETT = endotracheal tube; TIVA = total intravenous anesthetic; SGD = supraglottic airway; ESP = erector spinae plane block; MTP = mid-point transverse process to pleura block; PECS = pectoralis plane block; PIFB = pecto-intercostal fascial plane block.

	Overall
n	100
Age range (%)	
18-30	5 ( 5.0)
31-40	16 (16.0)
41-50	26 (26.0)
51-60	22 (22.0)
61-70	12 (12.0)
71-80	16 (16.0)
80+	3 ( 3.0)
Male (%)	3 ( 3.0)
BMI (%)	
<18	1 ( 1.0)
18-25	41 (41.0)
26-30	28 (28.0)
31-35	15 (15.0)
35-40	14 (14.0)
>40	1 ( 1.0)
Ethnicity (%)	
Afro-Caribbean	26 (26.0)
Asian	6 ( 6.0)
Mixed	1 ( 1.0)
White	60 (60.0)
Other	7 ( 7.0)
ASA (%)	
1	17 (17.0)
2	67 (67.0)
3	16 (16.0)
GA Technique (%)	
ETT+TIVA	13 (13.0)
SGD+TIVA	70 (70.0)
SGD+volatile	17 (17.0)
Blocks performed (%)	
None	33 (33.0)
ESP	10 (10.0)
ESP+MTP	8 ( 8.0)
Paravertebral	30 (30.0)
Paravertebral+PECS	1 ( 1.0)
Paravertebral+Serratus	1 ( 1.0)
PECS	12 (12.0)
PECS+MTP	1 ( 1.0)
PECS+PIFB	4 ( 4.0)
Surgical severity (%)	
Minor	12 (12.0)
Moderate	38 (38.0)
Major/Complex	50 (50.0)

**Figure 1:**
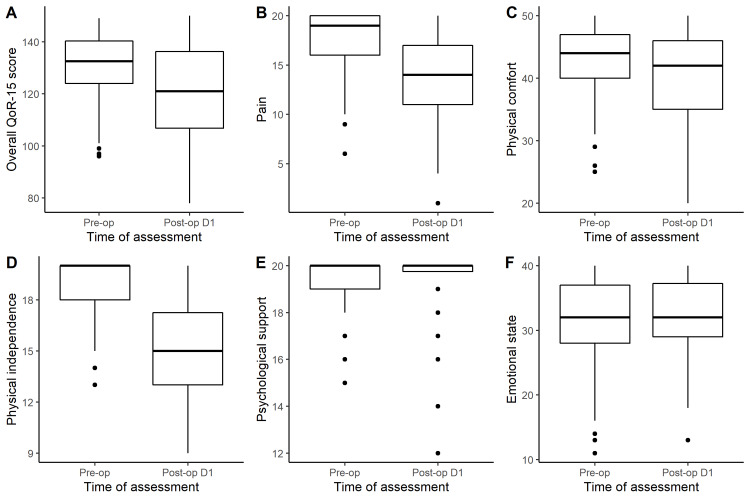
Boxplots of the preoperative and postoperative QoR-15 scores overall (A), and for each domain: Pain (B), physical comfort (C), physical independence (D), psychological support (E) and emotional state (F). QoR = Quality of recovery

The median (IQR) QoR-15 score at baseline was 133 (124-141), and on postoperative day 1, this was significantly decreased (median = 121, IQR: 106.75-136.25, Wilcoxon Signed-Rank Test, p<0.001) (Figure 1A).

Recovery for the cohort was overall good with 55/96 (57.3%) of patients recording postoperative day 1 QoR-15 scores of 118 or more. There was a positive correlation between preoperative QoR-15 scores and QoR-15 scores on postoperative day 1. The QoR-15 subdomains of pain, physical comfort and physical independence were all similarly reduced on postoperative day 1; however, psychological support and emotional state were unchanged (Figures 1B-1F).

In univariable linear regression analysis, volatile anesthesia was associated with lower postoperative QoR-15 scores compared to total intravenous anesthesia (TIVA), while the use of PVB or combination regional blocks was associated with higher postoperative QoR-15 scores compared to no regional blocks (Table 2).

**Table 2:** Associations between overall postoperative QoR-15 score and other variables. Univariable coefficients are reported alongside the adjusted coefficients from a multivariable model. Significant associations are shown in bold. QoR = Quality of recovery; TIVA = Total intravenous anesthesia; ASA = American Society of Anesthesiologists Physical Status grade; PECS = pectoralis plane block; ESP = erector spinae plane block

Dependent: Postoperative Day 1 QoR-15 Score		Coefficient (univariable)	Coefficient (multivariable)
Preoperative QoR-15 score		0.58 (0.33 to 0.83, p<0.001)	0.47 (0.23 to 0.72, p<0.001)
TIVA	TIVA	Reference	Reference
	Volatile	-9.11 (-18.08 to -0.15, p=0.046)	-6.22 (-14.71 to 2.28, p=0.149)
Blocks	None	Reference	Reference
	ESP	6.16 (-5.03 to 17.34, p=0.277)	5.88 (-5.90 to 17.66, p=0.323)
	Paravertebral	14.82 (6.97 to 22.67, p<0.001)	16.73 (7.65 to 25.81, p<0.001)
	PECS	10.77 (-0.88 to 22.42, p=0.070)	6.83 (-4.94 to 18.61, p=0.252)
	Combination	21.39 (11.73 to 31.05, p<0.001)	21.77 (12.78 to 30.76, p<0.001)
Age range	18-30	Reference	Reference
	31-40	-0.25 (-19.25 to 18.75, p=0.979)	2.17 (-14.80 to 19.14, p=0.800)
	41-50	-3.28 (-21.58 to 15.02, p=0.723)	3.38 (-12.93 to 19.70, p=0.681)
	51-60	4.23 (-14.25 to 22.70, p=0.650)	6.78 (-9.42 to 22.97, p=0.407)
	61-70	-0.00 (-19.84 to 19.84, p=1.000)	5.56 (-11.53 to 22.64, p=0.519)
	71-80	3.20 (-15.93 to 22.33, p=0.740)	8.82 (-7.91 to 25.55, p=0.297)
	80+	-19.33 (-45.29 to 6.63, p=0.142)	-7.66 (-30.42 to 15.11, p=0.505)
ASA	1	Reference	Reference
	2	-8.85 (-18.53 to 0.82, p=0.072)	-8.11 (-17.79 to 1.58, p=0.100)
	3	-10.23 (-22.37 to 1.91, p=0.098)	-5.69 (-18.06 to 6.69, p=0.363)
Severity	Minor	Reference	Reference
	Moderate	-0.44 (-11.92 to 11.03, p=0.939)	1.09 (-9.80 to 11.99, p=0.842)
	Major/Complex	-0.90 (-12.01 to 10.22, p=0.873)	-7.77 (-18.62 to 3.09, p=0.158)

**Figure 2:**
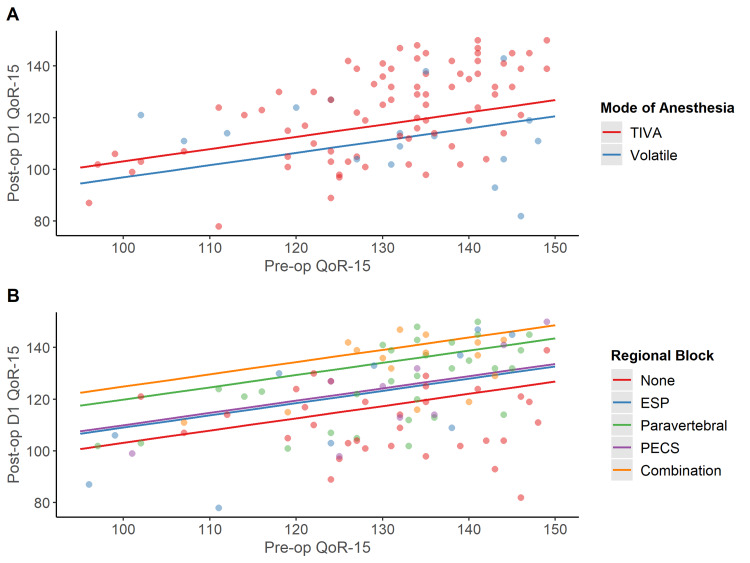
(A) Scatterplot illustrating the association between postoperative QoR-15 and the interaction between type of anesthetic received and preoperative QoR-15 from our multivariable linear regression model. (B) Scatterplot illustrating the association between postoperative QoR-15 and the type of regional block received from our multivariable linear regression. QoR = Quality of recovery; TIVA = Total intravenous anesthesia; ESP = Erector spinae plane block; PECS = Pectoralis nerve block

After covariate-adjustment in a multivariable model, significant associations between regional blocks received by patients and postoperative QoR-15 scores persisted (Table 2, Figure 2).

In our multivariable model, the use of PVB was associated with a 16.7 point higher overall QoR-15 score on Postoperative Day 1 compared to no block (95% Confidence Interval [CI]: 7.7-25.8, p <0.001); while the use of combination blocks was associated with a 21.8 point higher postoperative QoR-15 score compared to no block (95% CI: 12.8-30.8, p<0.001).

**Figure 3:**
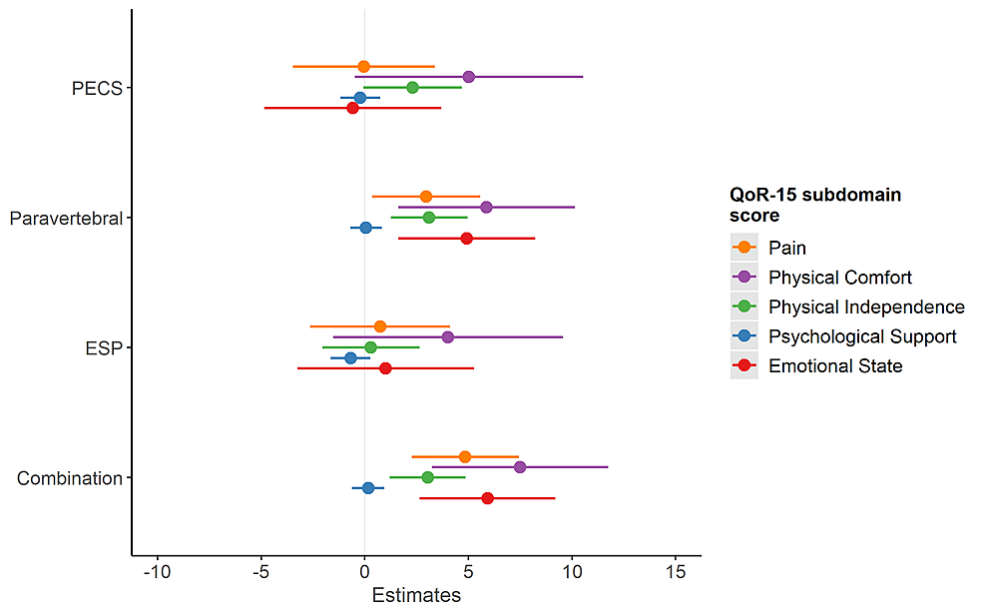
Associations between block type and QoR-15 subdomain outcomes. Compared to no block, and adjusting for patient age, ASA-grade, surgical severity, GA technique and preoperative scores, paravertebral and combination blocks were associated with increased postoperative scores for the following QoR-15 subdomains: pain, physical comfort, physical independence and emotional state. QoR = Quality of recovery; ESP = Erector spinae plane block; PECS = Pectoralis nerve block

Modelling each QoR-15 subdomain as a separate outcome, adjusting for the same model covariates as in the multivariable model above, PVB and combination blocks were associated with better pain, physical comfort, physical independence and emotional state scores on postoperative day 1, compared with receiving no regional anesthetic block (Figure 3).

## Discussion

Our data suggest that breast surgery patients receiving a PVB or a combination of regional blocks during surgery have higher QoR-15 scores on postoperative day 1, after adjustment for other factors. The estimated benefit associated with receiving either a PVB or a combination of blocks was both statistically and clinically significant based on previously published thresholds [2]. Furthermore, patients who received a combination of blocks (e.g. ESP + MTP or PECS + pecto-intercostal fascial block) tended to have better postoperative QoR-15 scores than the ones who only received an isolated PVB. In our cohort, higher postoperative QoR-15 scores were also reported by patients who received TIVA, but this association did not persist after adjustment for other covariates.

Our results are consistent with Abdallah et al., who demonstrated that, with the exception of psychological support, patients receiving PVB had better scores in all dimensions of the QoR questionnaire compared with patients in the control group [20]. Our results are also in agreement with the findings of Kamiya et al. and Barrington et al. who found that the PECS blocks alone were not significantly associated with an improved postoperative QoR score [21,22].

Though our sample size was small, we observed that patients who received a combination of blocks had, on average, better postoperative QoR-15 scores than those who only received an isolated PVB. One possible explanation for this finding may be that PVB are more difficult to perform, and in our cohort these blocks were performed by different operators with varying levels of experience. The ESP and MTP blocks have ultrasound landmarks that are easier to identify - (the transverse process [TP] and erector spinae muscle group in the case of ESP, and the midpoint between TP and pleura for MTP) and may be technically easier to perform for more junior practitioners and those who do not perform regional anesthesia techniques regularly. Also, it is possible that a combination of ESP and MTP was more likely to generate a “paravertebral by proxy” block than ESP alone, as local anesthetic injected in the intertransverse space may spread into the paravertebral space from these anatomically adjacent planes [23].

Anesthesiologists often do not follow patients up after surgery beyond the immediate postoperative period in the recovery room, and postoperative patient recovery has traditionally been assessed using blunt outcome measures of morbidity, mortality and re-hospitalization rates-events, which may occur sometime after the surgery itself [24]. Furthermore, while monitoring for postoperative mortality and major complications is important, these outcomes are rare for the majority of surgery. Therefore, clinicians require other means of assessing the quality of care they deliver. A patient’s ability to resume normal activities after surgery and discharge home, particularly after ambulatory or day surgery, is an important indicator of a successful perioperative experience [25]. In our cohort, all patients successfully completed their baseline QoR-15 questionnaire, and only a small number were lost to follow-up. This suggests that the QoR-15 can feasibly be used by clinicians for routinely measuring perioperative outcomes, especially in case-mixes where mortality and severe complications are expected to be low. QoR-15 takes on average 2.5 minutes to complete and has undergone extensive psychometric testing, performing well in all dimensions [18]. We now propose to measure QoR for other surgical specialties at our institution, and to administer the QoR-15 before surgery at the preoperative clinic (rather than the day of surgery).

We sought to evaluate the possible effects on QoR-15 scores on patients undergoing breast surgery at our institution, and the influence of RA and GA on this outcome. This is the first study to look at QoR-15 scores for a cohort of patients having multiple RA techniques reported for breast surgery, including ESP and MTP, which have previously not been reported. We have also included mastectomy and other more invasive surgeries for breast cancer in our study, which have been excluded in few of the previous studies.

Our study has some limitations. First, it was an observational study and therefore we cannot attribute causality of anesthetic techniques on outcomes. Second, it was conducted in a single tertiary institution and therefore may have limited generalizability to other clinical contexts; however our patient cohort was representative and diverse in terms of ethnicity, age, gender and comorbidities. Third, the anesthetic techniques used were not standardized, and there may be uncontrolled inter-operator differences in the conduct of anesthesia which may explain our findings. Finally, while we have evaluated a range of regional analgesia techniques used in multiple types of breast surgery, our findings are limited by small sample size, and our study may be underpowered to detect significant associations between other regional blocks and QoR-15 outcomes. Therefore, future studies with larger samples are warranted.

## Conclusions

The use of paravertebral and combination regional blocks in breast surgery is associated with better postoperative QoR scores. Paravertebral and combination regional blocks are further associated with improved QoR scores for pain, physical comfort, physical independence and emotional state, suggesting that these regional anesthetic techniques have multimodal effects beyond the mitigation of pain only. TIVA was associated with improved postoperative QoR scores in univariable analysis, but some of this effect may represent confounding and the association was no longer significant when other factors were accounted for. This may represent a weak association that our study was underpowered to detect. Future research should evaluate the effect of other regional analgesia techniques reported for breast surgery and QoR; larger sample sizes may be needed to detect associations between TIVA versus volatile anesthetic, and improved QoR.
